# Multiple drug transporters contribute to the brain transfer of levofloxacin

**DOI:** 10.1111/cns.13989

**Published:** 2022-10-17

**Authors:** Yuying Cen, Yuheng Shan, Jiahua Zhao, Xiaojiao Xu, Zhiyong Nie, Jiatang Zhang

**Affiliations:** ^1^ Medical School of Chinese PLA Beijing China; ^2^ Department of Neurology, The First Medical Centre Chinese PLA General Hospital Beijing China; ^3^ State Key Laboratory of Toxicology and Medical Countermeasures, Institute of Pharmacology and Toxicology Academy of Military Medical Sciences Beijing China

**Keywords:** blood–brain barrier, blood–cerebrospinal fluid barrier, drug transporters, levofloxacin, pharmacokinetics

## Abstract

**Aims:**

The aim of this study was to assess the influence of the major transporters at blood–brain barrier and blood–cerebrospinal fluid barrier on levofloxacin (LVFX) pharmacokinetics in rat. To explore the different effects of transporters on drug concentrations in cerebrospinal fluid (CSF) and brain extracellular fluid (ECF).

**Methods:**

High‐performance liquid chromatography coupled with microdialysis was used to continuously and synchronously measure unbound concentrations of LVFX in rat blood, hippocampal ECF, and lateral ventricle CSF for comprehensive characterization of brain pharmacokinetics. The role of transporters in the brain efflux mechanism of LVFX was analyzed in the absence and presence of various transporter inhibitors.

**Results:**

Following LVFX (50 mg/kg) administration, the unbound partition coefficient of LVFX in brain ECF and CSF (*K*_p,uu,ECF_ and *K*_p,uu,CSF_) were 34.0 ± 1.7% and 41.2 ± 2.4%, respectively. When probenecid was coadministered with LVFX, the AUC and the mean residence time (MRT) in rat blood increased significantly (*p* < 0.05). After MK571 intervention, 1.35‐fold and 1.16‐fold increases in *K*_p,uu,ECF_ and *K*_p,uu,CSF_ were observed, respectively (*p* < 0.05). Treatment with Ko143 increased the levels of LVFX in brain ECF. The difference in LVFX concentration in brain ECF and CSF was <3‐fold with or without treatment with transporter inhibitors.

**Conclusion:**

Efflux of LVFX from the central nervous system (CNS) involves multidrug resistance‐associated proteins (MRPs), breast cancer resistance protein (BCRP), and organic anion transporters (OATs). MRPs play an important role in mediating the brain/CSF‐to‐blood efflux of LVFX. LVFX concentrations in CSF can be used as a surrogate to predict the concentrations inside brain parenchyma.

## Graphical abstract

Efflux of levofloxacin from the CNS involves MRPs, BCRP, and OATs. MRPs are the major drug transporter limiting levofloxacin distribution in brain and its inhibitors can effectively improve blood–brain and blood–cerebrospinal fluid barrier permeability of levofloxacin. In addition, CSF can be used as a surrogate to predict the brain ECF concentration of levofloxacin.

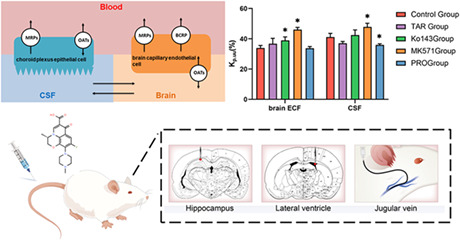

## 1 INTRODUCTION

Levofloxacin (LVFX) is a third‐generation fluoroquinolone with broad‐spectrum activity against Gram‐negative bacilli, Gram‐positive cocci, and atypical pathogens.^1^, ^2^ Current evidence suggests that levofloxacin has good cell permeability accounting for its high intracellular and tissue levels,^3^ which is essential for treating central nervous system (CNS) infections. In recent years, the number of patients with tuberculous (TB) meningitis resistant or intolerant to the first‐line anti‐TB drugs has substantially increased.^4^ Fluoroquinolones, including levofloxacin, are the most valuable second‐line anti‐TB agents.^5^ However, the safety and tolerability of LVFX remain a significant concern. Fluoroquinolone use may lead to CNS toxicity, which is reportedly related to the drug dose.^6^, ^7^ However, inadequate antimicrobial exposure may lead to treatment failure and increased mortality. Therefore, it is very important to elucidate mechanisms underlying the brain distribution of LVFX. Several evidences substantiate that the brain LVFX concentration is lower than in the plasma.^8^, ^9^ Even though brain barrier permeability increases during meningitis, a higher therapeutic dose is still required to maintain the drug concentration in the brain. Thus, the brain distribution of LVFX seems to be restricted clearly.

The blood‐cerebrospinal fluid barrier (BCSFB) located at the choroid plexus and the blood–brain barrier (BBB) located at the arteriole‐capillary‐venule level play an important role in limiting drug movement from the blood into the CNS.^10^, ^11^, ^12^ Studies have shown that several transporters expressed on cerebral capillary endothelial cells and monolayer ependymal cells of the choroid plexus may limit the levels of quinolones in the CNS and affect the drug distribution in blood, brain extracellular fluid (ECF), and cerebrospinal fluid (CSF).^13^, ^14^, ^15^, ^16^ We identified the transporters that may influence the distribution of quinolones by conducting a literature review^13^, ^17^, ^18^, ^19^ and studied their effects on the pharmacokinetic disposition of LVFX in the CNS. Indeed, the administration of specific inhibitors of transporters can inhibit their function and help explore the role of transporters in the BBB and BCSFB. These findings enable an in‐depth characterization of the impact of transporters on drug distribution in the CNS and promote the use of LVFX in treating CNS infections.

## 2 EXPERIMENTAL

### 2.1 Chemicals and reagents

Tariquidar and Ko143 were obtained from Sigma. MK571 sodium salt (purity ≥98.0%) was purchased from MedChem Express. Probenecid was obtained from Fisher Scientific. Levofloxacin (purity ≥98.0%) was purchased from CSNpharm. Dimethyl sulfoxide (DMSO) (purity ≥99.5%), polyethylene glycol 300 (PEG300), Tween‐80, phosphoric acid (85 wt%), and Chromatographic‐grade methanol were purchased from Acros Organics. All other chemicals were commercially available and of the highest grade available.

### 2.2 Apparatus and chromatographic conditions

The high‐performance liquid chromatography (HPLC) system consisted of an Agilent 1200 series degasser (G1322A), quaternary pump (G1312B), autosampler (G1367D), column oven (G1316B), and DAD detector (G1315C). The Agilent MassHunter Workstation software was used for instrument control and data acquisition. Chromatogram review and peak area integration were performed using Agilent MassHunter Qualitative Analysis 10.0 software. The chromatographic separation was performed using a Waters Sunfire C18 HPLC column (5 μm particle size, 150 mm × 4.6 mm, Walters). The column temperature was set at 35°C, and the detection wavelength was 294 nm. Optima retention of levofloxacin was achieved using an isocratic mobile phase of methanol: water containing 0.06% phosphoric acid in the ratio of 17:83 (v/v). Chromatographic separation was performed using a flow rate of 1.0 ml/min. The injection volume was 15 μl.

### 2.3 Method validation

Using artificial cerebrospinal fluid (ACSF) and Ringer's solution as solvents, the LVFX concentration required for calibration and quality control (QC) samples was obtained by appropriate step‐by‐step dilution. A calibration curve was constructed using the relationship between the analyte peak area and analyte concentration obtained in the range of 0.01–30 μg/ml. Linearity was assessed by a weighted (1/*x* ^2^) least squares regression analysis. The accuracy and precision of the method were evaluated by QC samples of high, medium, and low concentrations. Quantification of the concentration of QC samples was conducted three times on the same day to evaluate the intra‐day precision and accuracy. The same procedure was employed for 3 consecutive days to evaluate the inter‐day precision and accuracy of this method. The relative error (RE%) and relative standard deviation (RSD%) were used to evaluate the accuracy and precision, respectively. We evaluated the stability of LVFX in dialysates under short‐term (6 h) room temperature, low temperature (4°C), and freeze–thaw conditions (three cycles).

### 2.4 Animals

Adult (7‐week‐old) male Sprague Dawley rats (220–250 g; Vital River Laboratory) were used for the experiments. The rats were grouped and placed in polypropylene cages with wire mesh tops and maintained in a controlled environment (temperature, 22 ± 2°C; humidity, 55 ± 10%; standard light cycles of 12 h). After surgery, animals were housed individually. Standard diet and domestic quality mains water were available ad libitum. An acclimatization period of at least 3 days was observed before the animals were used in experiments. All animal experiments were approved by the Animal Use for Research and Education Committee of the General Hospital of Chinese People's Liberation Army.

### 2.5 Surgery

On the day of surgery, rats were anesthetized using inhaled isoflurane via a facemask. The rats were placed in a stereotaxic frame (Gene&I), and the body temperature was maintained with a homoeothermic blanket set at 37°C. A midline incision was made to expose the skull. According to the Paxinos and Watson atlas, two microdialysis intracerebral guide cannulae (Eicom Co.) were implanted into the hippocampus CA1 region (Hp‐CA1 coordinates: AP −4.16 mm; ML −2.2 mm; DV −2.9 mm) and lateral ventricle (LV coordinates: AP −1.4 mm; ML +2.2 mm; DV −3.4 mm). Fixed to the skull using a screw and dental cement. After 3 days of recovery, the rats were anesthetized with isoflurane again. An intravascular probe (TP‐100‐20; 20 mm, Eicom Co.) was implanted into the jugular vein toward the right atrium. After the blood microdialysis probe was positioned within the jugular vein, the pipeline was connected to perfuse with ACD solution (citric acid 3.5 mM; sodium citrate 7.5 mM; dextrose 13.6 mM) at a flow rate of 1.5 ml/min, and the Hamilton microinjection pump was used for continuous perfusion. After stopping isoflurane inhalation, the rats were allowed to wake up naturally on the homoeothermic blanket. The awake rats were placed in a device that allowed them to move freely. Animals were allowed to recover for an additional 24 h.

### 2.6 Microdialysis in vivo calibration

The in vivo microdialysis probe recovery of levofloxacin was determined based on reverse dialysis.^20^, ^21^ ACD solution and ACSF were used as perfusion fluids for blood and brain microdialysis, respectively. One hour after implantation of brain probes (AD‐6; 6 mm, Eicom Co.), blank perfusion fluids were changed to ACD solution with 5 μg/ml LVFX (for blood microdialysis) and ACSF solution with 0.5 microgram per milliliter LVFX (for brain microdialysis), which were injected at a rate of 2 μl/min through their respective probes. After a stabilization period of 1.5 h, six fractions were collected at 15 min intervals from each pipeline. After the recovery experiment, perfusion fluids containing LVFX were switched to blank perfusion fluids for pipeline washing. The in vivo recovery (*R*_dial_) of LVFX was calculated using the following equation:

$$R_{\text{dial}}=\frac{\left(C_{\text{perf}}-C_{\text{dial}}\right)}{C_{\text{perf}}}$$

$$C_{\text{perf}}=C_{\text{perfusate}}$$

$$C_{\text{dial}}=C_{\text{dialysates}}$$

All microdialysate concentrations (*C*_m_) from the hippocampal ECF and lateral ventricle CSF were converted to the unbound drug concentration (*C*_u_) at the target site using the following formula: *C*_u_ = *C*_m_/*R*_dial_.

### 2.7 In vivo microdialysis

#### 2.7.1 Animal grouping

A total of 32 rats were randomly divided into the following groups:

1. Control group, *N* = 12: vehicle solution + LVFX (50 mg/kg).
2. TAR group, *N* = 5: Tariquidar (8 mg/kg) + LVFX (50 mg/kg).
3. Ko143 group, *N* = 5: Ko143 (15 mg/kg) + LVFX (50 mg/kg).
4. MK571 group, *N* = 5: MK571 (20 mg/kg) + LVFX (50 mg/kg).
5. PRO group, *N* = 5: Probenecid (50 mg/kg) + LVFX (50 mg/kg).

Given that the solvents of intervention drugs were different, the control group was subdivided into four subgroups (*n* = 3). According to the preparation method of the transporter regulator, an equal volume of solvent was used as the control.

Group 1: vehicle solution of Tariquidar/Ko143 + LVFX (50 mg/kg).

Group 2: 1.25% sodium bicarbonate (NaHCO_3_) solution + LVFX (50 mg/kg).

Group 3: physiological saline alkalinized with NaOH (pH = 9.0–9.2) + LVFX (50 mg/kg).

Group 4: LVFX (50 mg/kg) alone.

Levofloxacin was freshly dissolved in isotonic saline and administered via tail vein injection. Thirty minutes before LVFX administration, the intervention drugs of each group were injected intraperitoneally. To reduce the possible effect of solvents on BBB or BCSFB permeability of LVFX, we prioritized water as the solvent. However, Tariquidar and Ko143 are insoluble in water, and a cosolvent must be used to prepare agents that animals can tolerate. According to the different physicochemical properties of these four inhibitors, the preparation of each transporter inhibitor solution in this experiment was as follows.

1. P‐glycoprotein (P‐gp) inhibitor Tariquidar^22^: Tariquidar was freshly dissolved in DMSO to 8 mg/ml and then diluted to 4 mg/ml with a solution containing 10% Tween‐80, 25% PEG300, and 65% sterile water.
2. Breast cancer resistance protein (BCRP) inhibitor Ko143^23^: Ko143 was freshly dissolved in DMSO to 15 mg/ml and then diluted to 7.5 mg/ml with a solution containing 10% Tween‐80, 25% PEG300, and 65% sterile water.
3. Multidrug resistance‐associated proteins (MRPs) inhibitor MK571^24^: MK571 sodium salt hydrate was dissolved in 1.25% (w/v) *aq* sodium hydrogen carbonate (NaHCO_3_) solution to obtain a concentration of 10 mg/ml.
4. Organic anion transporters (OATs) inhibitor probenecid^25^: Probenecid was dissolved in NaOH (pH = 9.0–9.2) alkalized physiological saline to obtain a concentration of 25 mg/ml.

The pipelines where the blood and brain probes were installed were perfused at the speed of 2 μl/min with ACD solution and ACSF, respectively. Two brain probes were inserted into guide cannulas of the target site and allowed to equilibrate for 1 h. Subsequently, the rats were treated according to their corresponding groups in the device where they could move freely. Microdialyses samples were collected every 15 min using a fraction collector (EFC‐96, Eicom Co.) for 3 h and stored at 4°C until analysis. Finally, rats were anesthetized and decapitated, and their brain was harvested. Brain sections were prepared using Leica CM1950 frozen slicer to verify the cannula's placement. Only animals with correct probe placement were included in this study.

### 2.8 Pharmacokinetics

The blood, brain ECF, and CSF pharmacokinetic parameters were calculated with non‐compartmental analysis using Phoenix WinNonlin version 8.2.0 (Pharsight Corporation). All microdialysis results were expressed as mean ± standard error of the mean (SEM). The area under the curve of unbound LVFX (AUC_u_) in blood, brain ECF, and CSF within 0–180 min was calculated using the linear trapezoidal method. The unbound partition coefficient of LVFX in brain ECF (*K*_p,uu,ECF_) and CSF (*K*_p,uu,CSF_) were obtained by the following equation:

$$K_{p,\mathrm{uu},\mathrm{ECF}}=\frac{{\mathrm{AUC}}_{u,\mathrm{ECF}}}{{\mathrm{AUC}}_{u,\text{blood}}}$$

$$K_{p,\mathrm{uu},\mathrm{CSF}}=\frac{{\mathrm{AUC}}_{u,\mathrm{CSF}}}{{\mathrm{AUC}}_{u,\text{blood}}}$$

### 2.9 Statistical analysis

Data were expressed as mean ± SEM. Statistical analysis and graphical presentation of data were performed using SPSS 20.0 (IBM Corporation) and GraphPad Prism® 6 software. The normality of continuous variables was checked by the Shapiro–Wilk normality test. The homogeneity equality of variance was checked by Levene's test. One‐way ANOVA was used to make control subgroup comparisons and analyze the pharmacokinetics of LVFX in the brain and blood without transporter inhibitors. When homoscedasticity was not supported, Welch's ANOVA was performed. Tukey's or Games‐Howell's post hoc test was used according to the results for homogeneity of variances. A comparison of the parameters between the control and experimental groups was performed using the Student's *t*‐test (two‐tailed). For non‐parametric data, the Kruskal–Wallis H‐test or Mann–Whitney U‐test was used as appropriate. Multiple comparisons were adjusted by the Bonferroni method. *p*‐values < 0.05 were statistically significant.

## 3 RESULTS

### 3.1 Validation of microdialysis probe placement in rat brain

**FIGURE 1:**
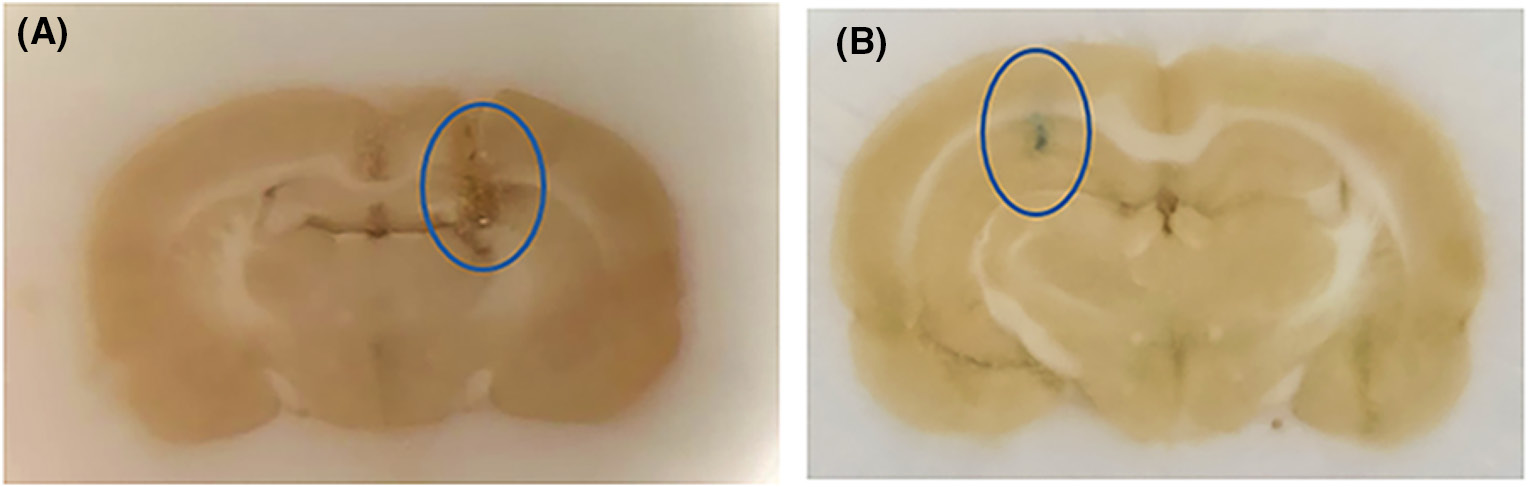
Validation of the placement of microdialysis probes. (A) Coronal brain section to validate the placement of the inserted microdialysis probe in the lateral ventricle. (B) Coronal brain section to validate the placement of the inserted microdialysis probe in the hippocampal.

The representative coronal brain section in Figure 1 shows that the scar caused by microdialysis probes reached the targeted regions for hippocampus and lateral ventricle.

### 3.2 Method validation

#### 3.2.1 Standard curve

The calibration curve for LVFX was linear ranging from 0.01 to 30 μg/ml. The regression equation of the calibration curve was *y* = 0.0141*x*−0.0097 (*R* ^2^ ≥ 0.998), where *x* is the peak area of the analyte and *y* is the concentration of the analyte.

#### 3.2.2 Accuracy, precision, and stability

The RSD% of intra‐and inter‐day precision was between 1.91% and 4.90%, while RE% of accuracy ranged from 0.99% to 5.14% (Table 1). The differences were within the acceptable range. The stability test of QC samples showed that the maximum deviation was 4.71% (Table 2). These findings showed that LVFX was stable under all conditions employed in this study.

**TABLE 1:** Intra‐day and inter‐day precision (RSD%) and accuracy (RE%) of the HPLC method for determination of LVFX in QC solutions (*n* = 3)

Analyst	Nominal concentration (μg/ml)	Intra‐day			Inter‐day		
		Observed concentration (μg/ml)	Precision (RSD%)	Accuracy (RE%)	Observed concentration (μg/ml)	Precision (RSD%)	Accuracy (RE%)
LVFX	0.3	0.299 ± 0.011	3.61	3.38	0.284 ± 0.014	4.90	5.14
	3	3.056 ± 0.107	3.50	1.85	3.056 ± 0.141	4.59	1.85
	30	29.410 ± 0.560	1.91	1.97	29.702 ± 0.649	2.18	0.99

**TABLE 2:** Stability of LVFX under different storage conditions (*n* = 3)

Storage condition	Nominal concentration (μg/ml)	LVFX	
		Calculated concentration (μg/ml) (mean ± SD)	RSD (%)
short term (room temp)‐6 h	0.3	0.293 ± 0.011	3.56
	3	3.098 ± 0.056	1.80
	30	30.608 ± 0.405	1.32
Storage for low temperature (4°C)‐12 h	0.3	0.294 ± 0.012	4.10
	3	3.055 ± 0.105	3.42
	30	29.844 ± 0.102	0.34
Freeze–thaw (3 cycles)	0.3	0.289 ± 0.008	2.55
	3	2.960 ± 0.140	4.71
	30	29.959 ± 0.417	1.39

### 3.3 Microdialysis probe calibration

In this study, the mean in vivo recoveries were 4.98 ± 0.42%, 4.17 ± 0.43%, and 32.31 ± 5.93% for probes in the hippocampus ECF, lateral ventricle CSF, and blood, respectively.

### 3.4 Microdialysis study of levofloxacin without intervention

#### 3.4.1 Effects of different vehicle solutions on the pharmacokinetics of levofloxacin in rats

Levofloxacin was detectable in blood and brain in all tests up to 180 min post‐dosing. No significant between‐subgroup differences in pharmacokinetic parameters were observed (Table 3). The experiment demonstrated that the vehicle solution of several intervention drugs and the intervention of intraperitoneal injection did not affect the distribution of LVFX in rats (Figure S1). To reduce the number of animals used, we analyzed the data of the above subgroups as a whole, representing the blank control without the intervention of transporter regulators.

**TABLE 3:** Pharmacokinetic parameters in blood, lateral ventricle CSF and hippocampus ECF after injection of LVFX (50 mg/kg) with different vehicle solution (mean ± SD, *n* = 3)

Parameters	Unit	Group 1	Group 2	Group 3	Group 4
Blood					
*C*_max_	μg/ml	10.37 ± 1.04	10.71 ± 0.32	9.78 ± 0.39	9.97 ± 0.59
*T*_max_	min	15	15	15	15
AUC_0–180_	min·μg/ml	620.12 ± 63.47	641.73 ± 43.30	560.62 ± 71.53	614.15 ± 29.43
AUC_0–∞_	min·μg/ml	658.53 ± 72.44	701.25 ± 78.66	604.44 ± 86.05	702.54 ± 55.14
*t*_1/2_	min	40.46 ± 5.68	50.47 ± 9.53	45.28 ± 6.27	60.59 ± 11.21
MRT	min	63.93 ± 4.74	73.91 ± 13.10	69.32 ± 10.34	86.20 ± 8.45
CSF					
*C*_max_	μg/ml	3.54 ± 0.15	3.86 ± 0.32	3.70 ± 0.14	3.48 ± 0.37
*T*_max_	min	15	15	15	15
AUC_0–180_	min·μg/ml	228.76 ± 18.68	248.13 ± 46.87	269.24 ± 47.49	259.97 ± 16.71
AUC_0–∞_	min·μg/ml	244.61 ± 23.80	269.40 ± 50.88	297.09 ± 66.98	280.81 ± 17.37
*t*_1/2_	min	38.03 ± 11.86	50.58 ± 8.78	45.76 ± 19.69	42.24 ± 5.60
MRT	min	70.79 ± 5.35	73.96 ± 7.80	81.82 ± 14.96	76.68 ± 2.63
Brain ECF					
*C*_max_	μg/ml	2.92 ± 0.52	2.72 ± 0.15	2.54 ± 0.17	3.05 ± 0.76
*T*_max_	min	15	15	15	15
AUC_0–180_	min·μg/ml	209.03 ± 23.69	194.11 ± 15.11	208.20 ± 37.05	215.84 ± 23.69
AUC_0–∞_	min·μg/ml	223.69 ± 31.20	215.99 ± 16.40	229.20 ± 49.39	236.66 ± 31.20
*t*_1/2_	min	39.82 ± 14.34	53.36 ± 9.24	49.78 ± 20.71	49.04 ± 17.85
MRT	min	74.12 ± 6.33	84.12 ± 6.64	83.96 ± 13.15	80.88 ± 19.58

Note

The specific grouping is shown in Section 2.7. Significant between‐group differences were not observed. Separated graphics used to present this table are in Figure S1.

#### 3.4.2 Pharmacokinetics of LVFX in rat brain and blood without transporter inhibitors

**FIGURE 2:**
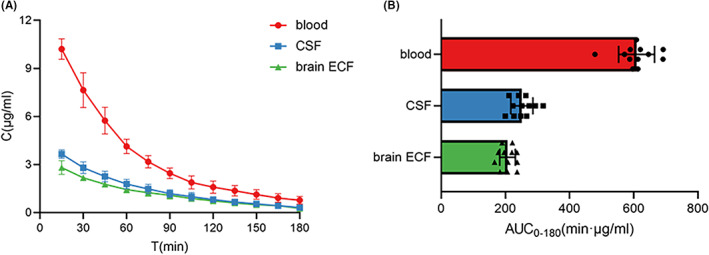
Pharmacokinetics of LVFX in brain and blood without transporter inhibitors (*N* = 12). (A) Concentration–time profiles in blood, CSF and brain ECF after tail vein administration of LVFX (50 mg/kg). (B) Area under the unbound concentration–time curve of in blood, CSF and brain ECF. Data are expressed as mean ± SD. Data used to plot these figures are provided in Table S1.

The highest concentration in blood and brain dialysate samples appeared in the first 15 min, followed by a gradual decrease in the drug concentration (Figure 2). There were significant differences in the *C*_max_, AUC_0–180_, and AUC_0–∞_ values of LVFX among blood, CSF and brain ECF. The specific pharmacokinetic parameters are described in Table 4. The blood levels were significantly higher than in the brain, while the LVFX concentration in CSF was slightly higher than in brain ECF. No differences in *t*_1/2_ and mean residence time (MRT) values were observed between blood, CSF and brain ECF, indicating a similar elimination rate of drugs in the three chambers. Without the intervention of transporter regulators, the *K*_p,uu,ECF_ and *K*_p,uu,CSF_ were 34.0 ± 1.7% and 41.2 ± 2.4%, respectively (Table S1).

**TABLE 4:** Key pharmacokinetic parameters of LVFX (50 mg/kg) with or without treatment with transporter inhibitors (mean ± SD, control group *N* = 12, experimental groups *N* = 5)

Parameters	Unit	Control group	TAR group	Ko143 group	MK571 group	PRO group
Blood						
*C*_max_	μg/ml	10.21 ± 0.70	10.38 ± 0.76	10.98 ± 0.77	10.10 ± 0.31	**17.48 ± 0.60** ^*^
*T*_max_	min	15	15	15	15	15
AUC_0–180_	min·μg/ml	609.15 ± 55.97	664.24 ± 58.46	664.03 ± 85.34	662.80 ± 34.53	1392.25 ± 109.58^*^
AUC_0–∞_	min·μg/ml	666.69 ± 75.69	706.28 ± 51.52	712.03 ± 116.29	704.81 ± 37.74	**1620.19 ± 149.81** ^*^
*t*_1/2_	min	49.20 ± 10.64	40.05 ± 6.51	42.80 ± 7.70	39.54 ± 4.79	**63.06 ± 9.61** ^*^
MRT	min	73.34 ± 11.90	69.17 ± 4.54	67.65 ± 11.12	69.70 ± 7.17	**94.87 ± 7.39** ^*^
CSF						
*C*_max_	μg/ml	3.65 ± 0.27	3.59 ± 0.28	**4.26 ± 0.32** ^*^	**5.46 ± 0.19** ^*^	**6.80 ± 0.24** ^*^
*T*_max_	min	15	15	15	15	15
AUC_0–180_	min·μg/ml	251.53 ± 34.25	234.01 ± 36.20	279.11 ± 10.90	**317.48 ± 25.71** ^*^	**499.57 ± 33.40** ^*^
AUC_0–∞_	min·μg/ml	272.98 ± 42.92	272.58 ± 17.85	296.10 ± 13.47	**351.77 ± 23.72** ^*^	**570.82 ± 51.44** ^*^
*t*_1/2_	min	44.15 ± 11.79	50.48 ± 18.64	41.76 ± 6.90	54.85 ± 7.43	**57.54 ± 7.55** ^*^
MRT	min	75.81 ± 8.70	79.98 ± 10.97	69.59 ± 4.33	75.82 ± 4.13	**87.92 ± 9.53** ^*^
*K*_p,uu,CSF_	%	41.2 ± 2.4	37.0 ± 1.3	42.4 ± 3.5	**47.9 ± 2.4** ^*^	**35.9 ± 0.8** ^*^
Brain ECF						
*C*_max_	μg/ml	2.71 ± 0.30	2.81 ± 0.45	**3.71 ± 0.25** ^*^	**4.21 ± 0.35** ^*^	**5.85 ± 0.18** ^*^
*T*_max_	min	15	15	15	15	15
AUC_0–180_	min·μg/ml	206.79 ± 24.37	245.86 ± 46.20	**257.01 ± 21.92** ^*^	**304.82 ± 13.57** ^*^	**469.61 ± 46.08** ^*^
AUC_0–∞_	min·μg/ml	226.38 ± 30.78	274.67 ± 53.79	**279.09 ± 30.31** ^*^	**337.59 ± 30.37** ^*^	**553.39 ± 80.48** ^*^
*t*_1/2_	min	48.00 ± 14.70	52.88 ± 9.83	44.41 ± 10.38	48.95 ± 15.11	**66.97 ± 16.83** ^*^
MRT	min	80.77 ± 11.59	86.08 ± 7.11	75.54 ± 9.07	80.89 ± 10.66	**96.46 ± 14.47** ^*^
*K*_p,uu,ECF_	%	34.0 ± 1.7	36.8 ± 3.5	**38.9 ± 2.4** ^*^	**46.0 ± 1.5** ^*^	33.7 ± 1.2

Note

The specific grouping is shown in Section 2.7. Significant data relative to the Control group were highlighted in bold, with the symbol * representing significance level: *p < 0.05.

### 3.5 Microdialysis study of levofloxacin in the presence of transporter inhibitors

#### 3.5.1 Effects of transporter inhibitors on plasma pharmacokinetic parameters of LVFX

**FIGURE 3:**
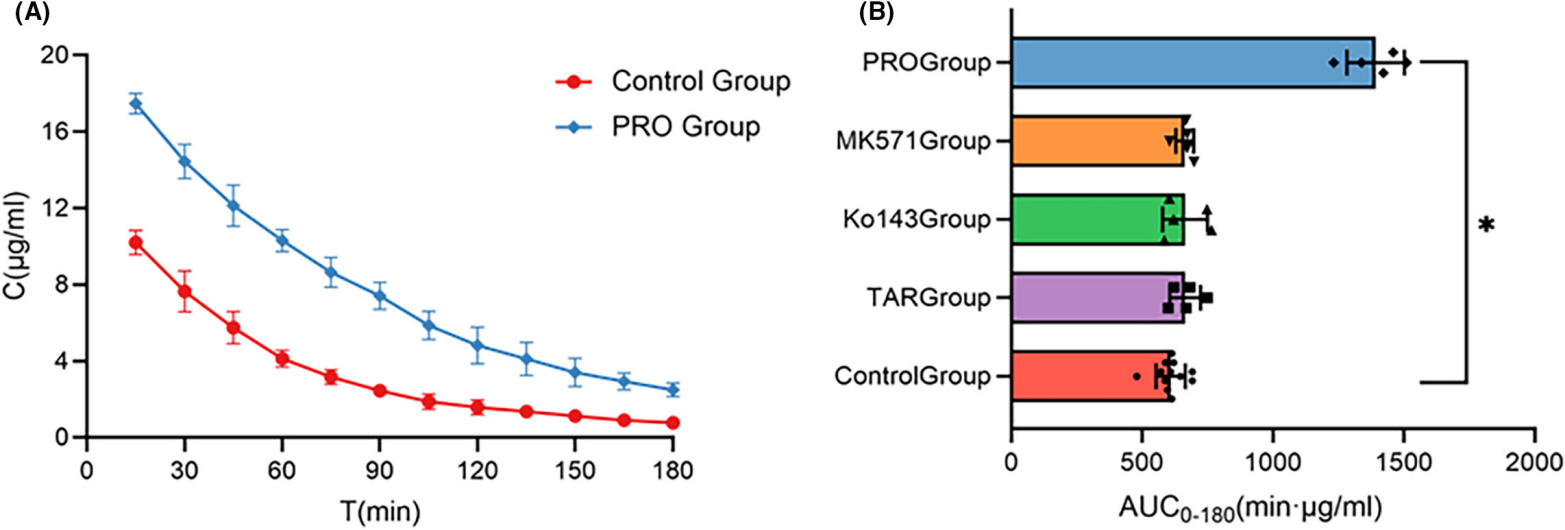
Effects of transporter inhibitors on plasma pharmacokinetic parameters of LVFX (control group *N* = 12, experimental groups *N* = 5). (A) Concentration–time profiles of LVFX in rat blood pretreated with probenecid. (B) Comparison of area under the unbound concentration–time curve of LVFX in rat blood in each group. Data are expressed as mean ± SD. *p < 0.05 relative to the Control group.

To assess the impact of transporter inhibitors on the peripheral levels of LVFX, plasma concentrations were compared between the experimental groups. We found that animals treated with Tariquidar, Ko143, or MK571 did not show any significant changes in the pharmacokinetics of LVFX in blood plasma, including *C*_max_, AUC, *t*_1/2_ and MRT (Table 4). Coadministration of probenecid altered the pharmacokinetic profile of LVFX in the blood (Figure 3). Compared with the control group, the AUC_0–180_ and AUC_0–∞_ of LVFX in the PRO group were 128.6% and 143.0% higher, and the *t*_1/2_ and MRT were 1.28 and 1.29 times longer, respectively. The *C*_max_ of LVFX in the PRO group was greater by 71.2%, while the peak time was not significantly altered. The above data suggested an increase in systemic drug levels.

#### 3.5.2 Effects of transporter inhibitors on the pharmacokinetics parameters of LVFX in CNS

**FIGURE 4:**
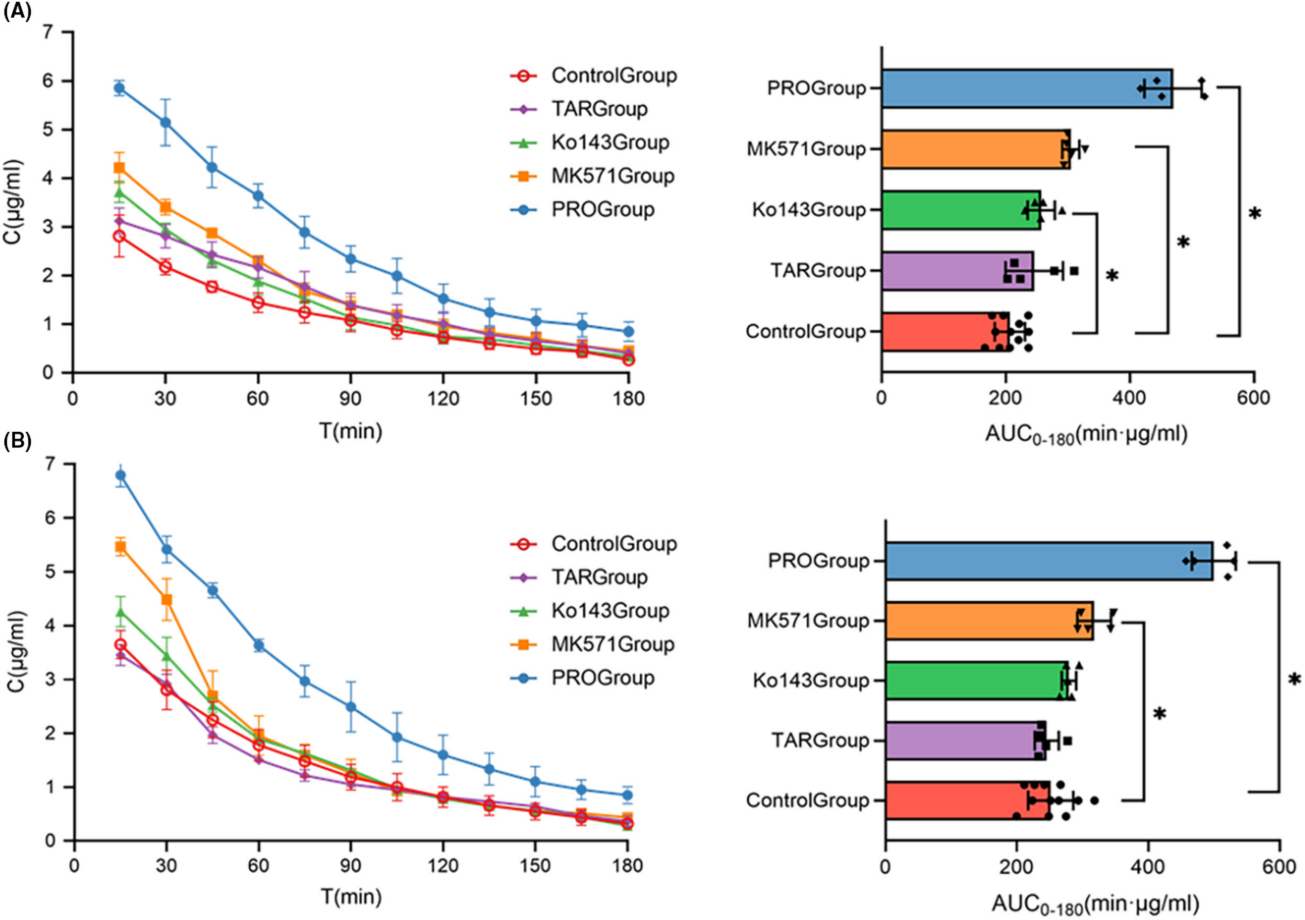
Effects of transporter inhibitors on the pharmacokinetics parameters of LVFX in CNS (control group *N* = 12, experimental groups *N* = 5). (A) Average unbound brain ECF concentration–time profiles of LVFX in control group and experimental groups. (B) Average unbound CSF concentration–time profiles of LVFX in control group and experimental groups. Data are expressed as mean ± SD. *p < 0.05 relative to the control group.

After administration of Tariquidar, there was no significant change in LVFX in the rat brain. The intervention of Ko143 increased the AUC value and concentration of LVFX in brain ECF. Although the drug concentration in CSF increased slightly, it did not alter the AUC value of this chamber. MK571 improved penetration of LVFX into CNS. The *C*_max_, AUC_0–180_, and AUC_0–∞_ values in brain ECF and CSF were higher than in the control group. Treatment with MK571 increased the *C*_max_ and AUC_0–180_ in brain ECF by 1.55‐fold and 1.47‐fold, respectively, and *C*_max_ and AUC_0–180_ in CSF by 1.50‐fold and 1.26‐fold, respectively. In rats pretreated with probenecid, we observed increased brain concentrations and the prolongation of *t*_1/2_ (Figure 4).

To evaluate the effect of transporter inhibitors on LVFX penetration into CNS, the *K*_p.uu.ECF_ and *K*_p.uu.CSF_ were calculated to represent the net transport of BBB and BCSFB (Table 4). The results suggested that inhibition of MRPs and BCRP led to increased transport of LVFX through the BBB. Only the intervention of MK571 could improve the permeability of BCSFB. When LVFX was combined with MK571, *K*_p,uu,ECF_ rose from 34.0 ± 1.7% to 46.0 ± 1.5%, *K*_p,uu,CSF_ from 41.2 ± 2.4% to 47.9 ± 2.4% (*p* < 0.05). Moreover, 1.35‐fold and 1.16‐fold increases in *K*_p,uu,ECF_ and *K*_p,uu,CSF_ were observed, respectively. Probenecid increased the brain and plasma LVFX concentrations. However, the plasma pharmacokinetic parameters changed more significantly, which causes a decrease in permeation.

#### 3.5.3 Relationship between the LVFX levels in CSF and ECF

**FIGURE 5:**
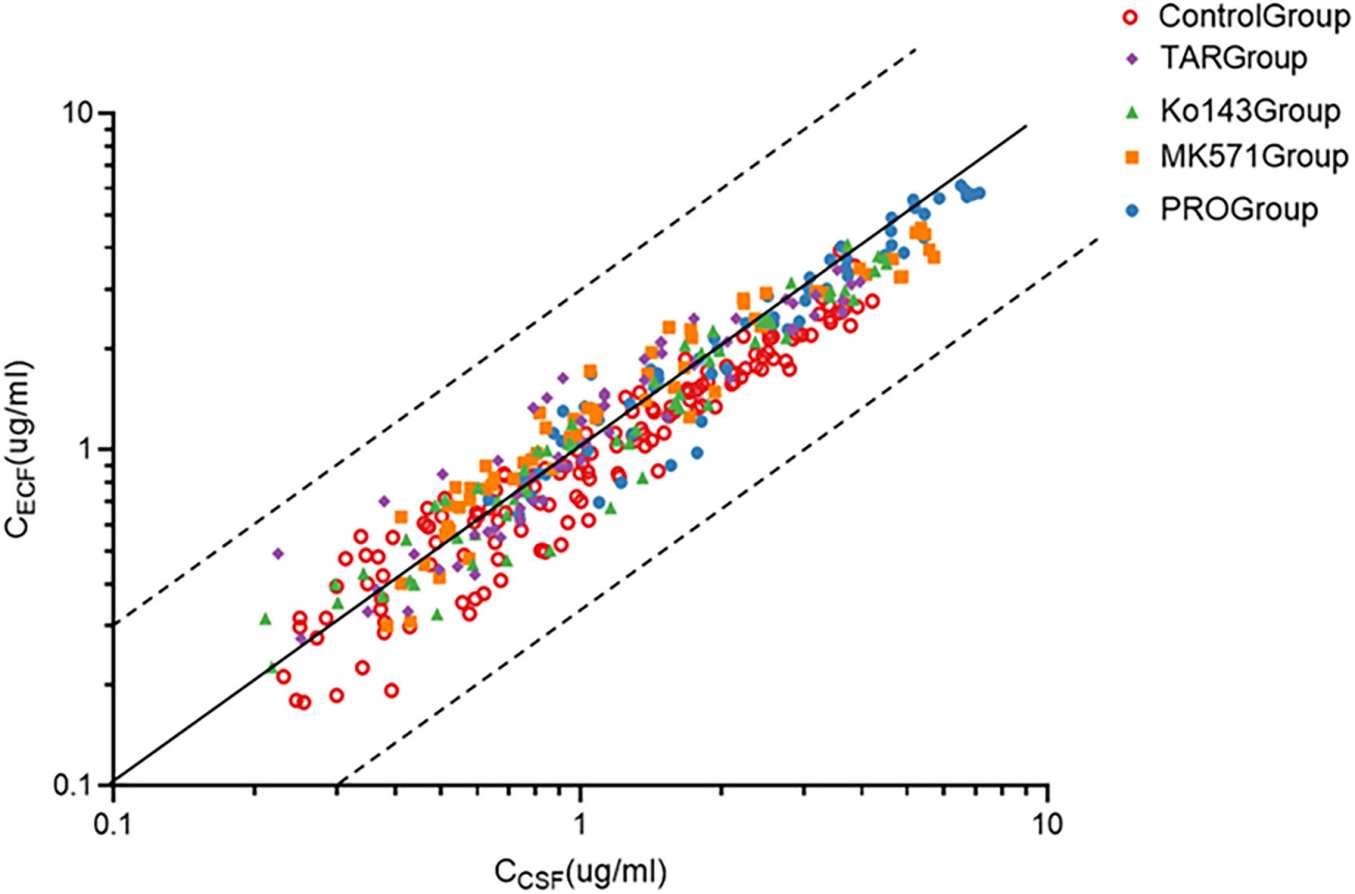
Relationship between CSF concentration and unbound brain ECF concentration. The solid line passing through the origin represents the line of unity ±3‐fold (dashed lines).

We observed a consistent change in brain ECF and CSF levels during the comparison of the pharmacokinetic parameters and concentration–time curve of LVFX between the control group and the experimental group. The difference in LVFX concentration and AUC value between brain ECF and CSF was <3 times (Figure 5) with or without treatment with transporter inhibitors which substantiated that CSF could be used as a marker for evaluating LVFX brain distribution.

## 4 DISCUSSION

**FIGURE 6:**
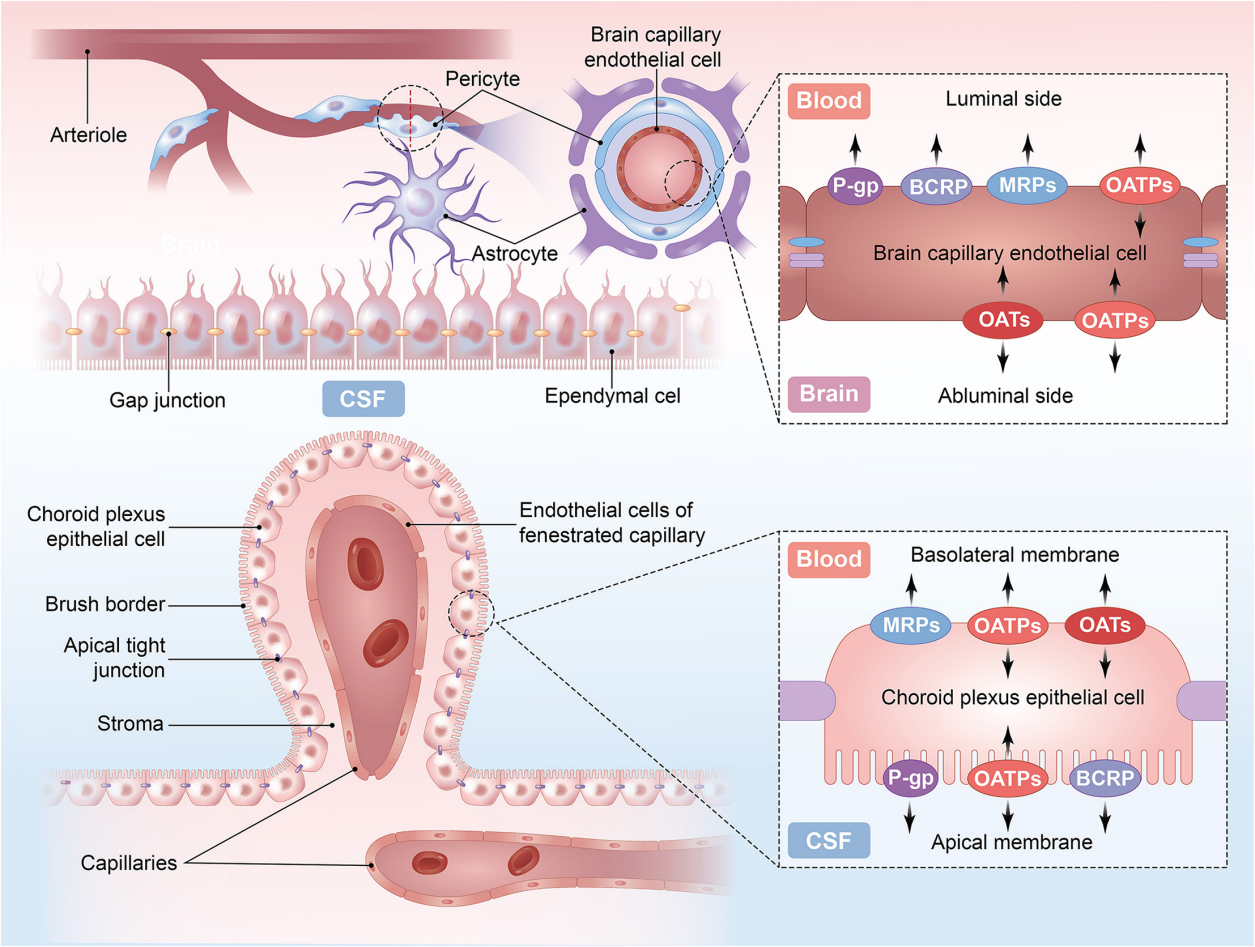
Putative localization of drug transporters proteins associated with LVFX on brain capillary endothelial cells and the choroid plexus epithelial cells.

Importantly, the present study's findings can be harnessed for planning and formulating effective treatment regimens with levofloxacin for meningitis or encephalitis. Here, we briefly summarize the present studies of the distribution and direction of the main transporters of quinolones in the rodent brain (Figure 6).

According to the free drug hypothesis, the passage of drugs across the BBB is restricted to the unbound fraction. The remaining drug fraction is partially bound to proteins in plasma.^26^, ^27^ Therefore, free drugs in brain tissue represent the fraction that works on target organs. In our study, the highest concentration of LVFX appeared in the dialysate sample within the first 15 min, indicating its rapid blood absorption and brain penetration. During 180‐min microdialysis sampling, drug concentrations in the blood were overall significantly higher than in the brain, indicating that LVFX is restricted to the brain. *K*_p.uu.ECF/CSF_ parameter is the most relevant parameter to interpret the extent of BBB or BCSFB transport, indicating the dominant mode of drug distribution across the blood–brain or blood–CSF barrier.^28^, ^29^ Findings observed in the blank control group in our study were consistent with the literature.^9^, ^14^ The *K*_p,uu,CSF_ value was slightly higher than *K*_p,uu,ECF_ value, likely attributable to the physiological and structural differences between barriers. Given that capillary endothelial cells are more tightly packed than choroid plexus epithelial cells, the BBB restricts diffusional and paracellular transport more than the BCSFB.^30^ If active transport is not involved, unbound drug molecules reach a balance between blood and brain.^29^ Although the current experiment was performed under non‐steady‐state conditions, the *K*_p,uu,ECF_ and *K*_p,uu,CSF_ values indicated the involvement of active efflux in the route of LVFX through the BBB and BCSFB.

Unlike the other groups where there was no alteration in the plasma pharmacokinetic parameters of LVFX, the intervention of probenecid significantly increased LVFX levels in the blood. LVFX is mainly eliminated by glomerular filtration and tubular secretion. The increased blood exposure caused by probenecid was most likely due to inhibition of OATs at the proximal tubules in the kidney (clearance organs), resulting in decreased renal secretion. Consistent with the results of the previous study, probenecid significantly altered the renal clearance of OATs substrates prolonging the elimination *t*_1/2_ of the drugs.^31^, ^32^

The experimental data suggested that LVFX may have low or no affinity for P‐gp and inhibiting the efflux transporter would not affect the drug concentration in blood. This finding is consistent with previous observations in knockout mice.^14^ In the MK571 group and Ko143 group, inhibitors of MRPs and BCRP increased the brain concentration but did not change plasma levels. According to the concept of overall intrinsic clearance, reduced efflux transporter activity can increase drug exposure in the brain. However, in target tissues with relatively small distribution volumes, the change in local drug exposure does not always lead to a change in plasma drug concentration.^33^, ^34^

It has been established that the cellular localization of transporters in brain endothelial cell monolayer and choroid plexus epithelial cell monolayer is crucial in determining the direction of drug transport. In the cerebral endothelium, BCRP is expressed on the luminal side of the plasma membrane, while in the choroid plexus epithelium, BCRP is expressed in the apical membrane.^35^, ^36^, ^37^ Therefore, localization of BCRP in the BBB suggests that it could actively efflux and transport the corresponding substrate to the luminal side, reducing the drug concentration in brain ECF. The localization of BCRP in the BCSFB suggests that it would facilitate but not restrict drug transport to CSF. It is worth noting that we did not observe any significant changes in the AUC value of levofloxacin in the CSF. BCRP is one of the most abundant transporters in the BBB of humans, monkeys, and rodents. However, its function in BCSFB remains questionable.^38^, ^39^ Kodaira et al.^40^ did not observe a decrease in the concentration of BCRP‐specific substrate in the CSF of BCRP knockout mice, consistent with our findings. A quantitative study of BCRP by Uchida et al.^41^ also indicated low BCRP protein expression in the rat choroid plexus cells. We speculate that active transport may not be the main route of levofloxacin penetration from plasma into CSF.

Multidrug resistance‐associated proteins are classified under the ABCC family. MRP1 to 5 have been identified to play a role in the brain drug transport system.^42^ The main subtype expressed in rat brains is MRP1. MRPs are located at the apical plasma membrane of brain capillary endothelial cells and the basolateral side of choroid plexus epithelium, conferring transport to the blood side.^35^, ^41^, ^43^ The location of MRPs determines its relevance in restricting brain access to corresponding substrates. Inhibition of MRPs weakens the barrier function leading to a significant increase in the drug concentration in the CNS. Most available studies used probenecid as an inhibitor of OATs (mainly OAT3).^44^, ^45^ However, probenecid is not a specific transporter inhibitor. It also has a certain effect on OATs, OATPs (organic anion‐transporting polypeptides), and MRPs.^46^, ^47^, ^48^ OATs and OATPs are bidirectional transporters,^49^ and it is difficult to evaluate their net efflux effect only by probenecid inhibition. Our results suggest that OATs and OATPs showed an overall efflux effect, transporting levofloxacin from the brain to the blood.

We calculated the *K*_p,uu,ECF_ and *K*_p,uu,CSF_ values in experimental groups (Table 4). After comparison, it was found that only MK571 could effectively change the pharmacokinetic parameters of LVFX in CNS and significantly increase the drug concentration in CSF and brain ECF. BCRP inhibitor also slightly improved LVFX levels in the brain.

Clinically, it is very difficult to obtain brain ECF samples. The drug unbound concentration in CSF is commonly used as the surrogate of the unbound drug in brain ECF.^50^ It has long been thought that the drug concentration in CSF was in equilibrium with the concentration of ECF in the brain, given the leaky ependymal cell monolayer between the brain and CSF interface.^51^, ^52^, ^53^ However, contrasting findings have been reported in the literature.^36^, ^54^, ^55^ In addition to considering the anatomical differences between brain ECF and CSF compartments, the different localization of transporters (mainly P‐gp and BCRP) on BBB and BCSFB greatly impacts drug transport. Our study suggested that LVFX may have a low affinity for P‐gp and MRPs are its major transporters for penetration into CNS. The present experimental findings indicate that the ECF concentration of LVFX is an appropriate surrogate of the pharmacological target concentration in the CNS.

## 5 CONCLUSION

In conclusion, this study demonstrated the pharmacokinetics of LVFX in rats and studied the role of major transporters in affecting its distribution in the brain. Our results provide compelling evidence that MRPs are the major efflux transporter limiting the distribution of LVFX in the CNS. Inhibition of MRPs could significantly increase LVFX exposure in the brain. Additionally, CSF can be used as a surrogate to predict the brain ECF concentration of LVFX.

## AUTHOR CONTRIBUTIONS

CYY performed all experiments and wrote the manuscript; SYH, ZJH, and XXJ helped with the animal experiments study; ZJT and NZY conducted and supervised the whole experiments. All authors are in agreement with the manuscript and have approved the manuscript for submission.

## CONFLICT OF INTEREST

There are no conflicts of interest to declare.

## Supporting information


Figure S1

Table S1

## Data Availability

The data that support the findings of this study are available from the corresponding author on reasonable request.
