# Supplementary material for: Multiple drug transporters contribute to the brain transfer of levofloxacin
Source: CNS Neurosci Ther. 2022 Oct 17;29(1):445–57. doi: 10.1111/cns.13989 (PMC9804084; doi:10.1111/cns.13989)
Supplement: Supplementary file 1 — Figure S1 [file CNS-29-445-s001.zip › Supplementary Figure 1.docx]

**Supplementary Figure 1**: Concentration-time profiles in blood, CSF and brain ECF after injection of LVFX (50mg/kg) with different vehicle solution. Data was showed in mean ± SD (n=3).

The specific grouping is shown in 2.7.

Significant between-group differences were not observed.
