# Supplementary material for: Multiple drug transporters contribute to the brain transfer of levofloxacin
Source: CNS Neurosci Ther. 2022 Oct 17;29(1):445–57. doi: 10.1111/cns.13989 (PMC9804084; doi:10.1111/cns.13989)
Supplement: Supplementary file 2 — Table S1 [file CNS-29-445-s002.docx]

**Supplementary Table 1** Comparison of pharmacokinetic parameters in blood, CSF and brain ECF after injection of LVFX (50mg/kg). (mean ± S.D., N=12).

| **Parameters** | **Unit** | **Blood** | **CSF** | **Brain ECF** | ***P* value** |
| --- | --- | --- | --- | --- | --- |
| **C_max_** | µg/ml | 10.21±0.70 | 3.65±0.27 | 2.71±0.30 | **0.000*** |
| **AUC_0-180_** | min·µg/ml | 609.15±55.97 | 251.53±34.25 | 206.79±24.37 | **0.000*** |
| **AUC_0-∞_** | min·µg/ml | 666.69±75.69 | 272.98±42.92 | 226.38±30.78 | **0.000*** |
| **t_1/2_** | min | 49.20±10.64 | 44.15±11.79 | 48.00±14.70 | 0.591 |
| **MRT** | min | 73.34±11.90 | 75.81±8.70 | 80.77±11.59 | 0.150 |
| **K_p,uu,CSF/ECF_** | % |  | 41.2±2.4 | 34.0± 1.7 | **0.000*** |

† The C_max_, AUC_0-180_ and AUC_0 - ∞_ values of blood, CSF and brain ECF were significantly different during post hoc comparison.

* *P* < 0.05 was considered statistically significant
